# Alterations in Gut Microbiota by Statin Therapy and Possible Intermediate Effects on Hyperglycemia and Hyperlipidemia

**DOI:** 10.3389/fmicb.2019.01947

**Published:** 2019-09-04

**Authors:** Jiyeon Kim, Heetae Lee, Jinho An, Youngcheon Song, Chong-Kil Lee, Kyungjae Kim, Hyunseok Kong

**Affiliations:** ^1^College of Pharmacy, Sahmyook University, Seoul, South Korea; ^2^College of Pharmacy, Chungbuk National University, Cheongju, South Korea; ^3^College of Animal Biotechnology and Resource, Sahmyook University, Seoul, South Korea

**Keywords:** atorvastatin, rosuvastatin, gut microbiota, IL-1β, TGFβ1, short-chain fatty acid

## Abstract

Dysbiosis of the gut microbiota is a contributing factor for obesity-related metabolic diseases such as hyperglycemia and hyperlipidemia. Pharmacotherapy for metabolic diseases involves the modulation of gut microbiota, which is suggested to be a potential therapeutic target. In this study, the modulation of gut microbiota by statins (cholesterol-lowering drugs: atorvastatin and rosuvastatin) was investigated in an aged mouse model of high-fat diet-induced obesity, and the association between gut microbiota and immune responses was described. Atorvastatin and rosuvastatin significantly increased the abundance of the genera *Bacteroides*, *Butyricimonas*, and *Mucispirillum.* Moreover, the abundance of these genera was correlated with the inflammatory response, including levels of IL-1β and TGFβ1 in the ileum. In addition, oral fecal microbiota transplantation with fecal material collected from rosuvastatin-treated mouse groups improved hyperglycemia. From these results, the effect of statins on metabolic improvements could be explained by altered gut microbiota. Our findings suggest that the modulation of gut microbiota by statins has an important role in the therapeutic actions of these drugs.

## Introduction

Obesity-related metabolic diseases including hyperlipidemia, hypertension, and type 2 diabetes (T2D) are prevalent, global health burdens (Wang et al., 2008). Lifestyle, including a lack of physical exercise and a Westernized diet, is a main contributing factor for the prevalence of metabolic diseases (Lee S.E. et al., 2018). Metabolic diseases are associated with an increased mortality risk in the elderly population; in particular, hyperlipidemia is an important risk factor for cardiovascular disease (Isomaa et al., 2001; Felix-Redondo et al., 2013).

Recently, American College of Cardiology/American Heart Association (ACC/AHA) recommended statins as a first-line treatment of hyperlipidemia, and statins are currently the most widely used treatment for hyperlipidemia (Safeer and Lacivita, 2000; Stone et al., 2014). Statins work by inhibiting 3-hydroxy-3-methylglutaryl-coenzyme A (HMG-CoA) reductase, an important enzyme involved in the cholesterol synthesis pathway (Stancu and Sima, 2001). Moreover, statins induce the expression of low-density lipoprotein (LDL) receptors and promote the removal of LDL cholesterol from the blood (Young and Fong, 2012). Furthermore, statins have been reported to have anti-inflammatory and immunomodulatory effects (Lee et al., 2016).

Gut microbiota is an important environmental factor for the regulation of energy homeostasis and immune system (Wu and Wu, 2012; Sanz et al., 2015). Modulation of gut microbiota improves the outcome of various diseases, including metabolic syndrome and inflammatory diseases, and influences pharmacotherapy (Wilson and Nicholson, 2009; Karkman et al., 2017). Recently, various interventions such as dietary supplement (Lee and Ko, 2016; Talsness et al., 2017), prebiotics or probiotics (Delzenne et al., 2011; Markowiak and Slizewska, 2017), and drugs (Shin et al., 2013; Lee and Ko, 2014) were reported to alter the composition of gut microbiota, which has an important role in the alleviation of metabolic diseases. In recent studies, statin therapy was found to affect the composition of gut microbiota (Khan et al., 2018; Liu et al., 2018). Nolan et al. (2017) showed that rosuvastatin influenced gut microbiota and significantly increased the abundance of the family *Lachnospiraceae*, and the genera *Rikenella* and *Coprococcus* in HFD-fed C57BL/6 mice.

The objectives of this study were (1) to identify the effects of statins on gut microbiota and metabolic improvements and (2) to investigate the correlation between significant changes in microbiota induced by statins and inflammatory regulation.

## Materials and Methods

### Animals and Experimental Protocol

Male 4-week-old C57BL/6N mice were purchased from Samtako Co., Ltd (Osan, South Korea) and acclimated to laboratory conditions for 1 week, in which the animals were housed with free access to water and food in a temperature–humidity-controlled animal facility under a 12-h light–dark cycle at 22 ± 2°C and 55 ± 5% humidity. Mice were fed either a 45% kcal high-fat diet (HFD) (FeedLab, Inc., Guri, South Korea) to induce metabolic disorders such as obesity, hyperglycemia, and hyperlipidemia, or a regular diet (RD) (Purina Korea, Inc., Seoul, South Korea) for 39 weeks. Atorvastatin (HFD-Ator: 10 mg/kg of body weight, *n* = 6) or rosuvastatin (HFD-Rosu: 3 mg/kg of body weight, *n* = 6) was administered every day during the HFD for the last 16 weeks. A RD-fed mice group (*n* = 6) and a HFD-fed mice group (*n* = 6) were included as normal and disease controls, respectively. All experimental procedures were performed in accordance with ethical guidelines issued by the Institutional Animal Care and Use Committee (IACUC) of Sahmyook University for the care and use of laboratory animals (SYUIACUC 2015001).

### Metabolic Measurements

Body weight and serum glucose levels in mice were monitored every 2nd week. Mice were fasted overnight (12 h), blood samples were taken from a tail cut, and serum glucose levels were measured using the Accu-Chek Performa system (Roche, Switzerland). Intraperitoneal glucose tolerance testing (IPGTT) was performed 16 weeks after statin (Atorvastatin; Ator, Rosuvastatin; Rosu) administration. After overnight fasting (12 h), mice were intraperitoneally injected with glucose solution [2 g/kg of body weight, in phosphate-buffered saline (PBS)] and blood samples were obtained from the tail vein 0, 30, 60, 90, and 120 min after glucose injection. At the end of the experimental period, mice were sacrificed under ether anesthesia and blood was collected *via* cardiac puncture. Blood sample were centrifuged at 10,000 rpm for 5 min to isolate serum. Serum was prepared to determine levels of total cholesterol, LDL, apolipoprotein A-1 (ApoA-1), and apolipoprotein B (ApoB) using a biochemical analyzer (AU480, Beckman Coulter, United States).

### Immunomodulatory Biomarkers

Ileum tissue from each group were immediately frozen in liquid nitrogen and stored at -70°C to determine levels of gene transcripts. The expression of interleukin-1β (IL-1β; forward primer: 5′-CAGGATGAGGACATGACACC-3′, reverse primer: 5′-CTCTGCAGACTCAAACTCCAC-3′), interleukin-6 (IL-6; forward primer: 5′-GTACTCCAGAAGACCAGAGC-3′, reverse primer: 5′-TGC TGG TGA CAA CCA CGG CC-3′), transforming growth factor β 1 (TGFβ1; forward primer: 5′-GCGGACTACTATGCTAAAGAGG-3′, reverse primer: 5′- GTAGAGTTCCACATGTTGCTCC-3′), interleukin-4 (IL-4; forward primer: 5′- GAGCCATATCCACGGATGCGACAA-3′, reverse primer: 5′- CATGGTGGCTCAGTACTACGAGTA-3′), and interleukin-10 (IL-10; forward primer: 5′-TGGCCACACTTGAGAGCTGC-3′, reverse primer: 5′-TTCAGGGATGAAGCGGCTGG-3′) was investigated in ileum tissue. Total RNA was extracted using a RiboEx^TM^ (GeneAll, Korea). RNA was then quantified by reading the absorbance at 260 nm. cDNA was synthesized using a HyperScript^TM^ RT premix (GeneAll, Korea). To quantify the level of mRNA expression, SYBR^®^ Green PCR Master Mix (Applied Biosystems, United States) and a StepOnePlus^TM^ real-time PCR system (Applied Biosystems, United States) were used. GAPDH was used as an internal control.

### Gut Microbiota Analysis

Total DNA was extracted using the PowerSoil DNA Isolation Kit (MO BIO Laboratories, Inc., United States) from cecum samples, which included fecal materials. Partial sequences of 16S rRNA genes were amplified based on the 16S rRNA amplification protocol from the Earth Microbiome Project (Gilbert et al., 2010). 16S rRNA genes were amplified using the 515F/806R primer set, which includes an adapter sequence for amplification of the V4 region (515F forward primer: 5′-TCG TCG GCA GCG TCA GAT GTG TAT AAG AGA CAG **GTG CCA GCM GCC GCG GTA A**-3′; 806R reverse primer: 5′-GTC TCG TGG GCT CGG AGA TGT GTA TAA GAG ACA **GGG ACT ACH VGG GTW TCT AAT**-3′). To attach the dual indices and adapter to amplified polymerase chain reaction (PCR) products, an index PCR was additionally performed using an AmpONE^TM^ α-Pfu DNA polymerase (GeneAll, Korea) and Nextera^®^ XT Index Kit v2 (Illumina, United States). PCR products after amplification and attachment were purified using the Expin^TM^ PCR SV (GeneAll, Korea). Partial bacterial 16S rRNA genes were amplified using the MiSeq Reagent Kit V3 (600 cycles) and MiSeq platform (Illumina, United States) at KoBioLabs, Inc.

Prior to analysis of 16S rRNA sequences, BCL files were converted into raw FASTQ files including read1, index, and read2 sequences using CASAVA-1.8.2 (Illumina). After preprocessing (quality filtering and trimming steps using FASTX-Toolkit) (Gordon and Hannon, 2010), sequences were assigned to operational taxonomic units (OTUs, 97% identity), and representative sequences were selected using QIIME 1.7.0 software (Caporaso et al., 2010). Next, taxonomic composition, alpha diversity (rarefaction curve and Shannon index of bacterial diversity) and beta diversity (PCoA of UniFrac distances) were analyzed. LDA effect size (LEfSe) was used to estimate taxonomic abundance and characterize differences between groups (Segata et al., 2011). Hierarchical clustering of the top ten most abundant bacterial genera by groups was performed using Spearman’s rank correlation, and a heat map was generated using MultiExperiment Viewer (MEV) software (v4.8.1). Next-generation sequencing (NGS) data are available at^1^.

The relative abundances of four bacterial genera were confirmed using SYBR^®^ Green PCR Master Mix (Applied Biosystems, United States) and a StepOnePlus^TM^ real-time PCR system (Applied Biosystems, United States). Genus-specific primer sets for Eubacteria (UniF340; forward primer: 5′-ACTCCTACGGGAGGCAGCAGT-3′, UniR514; reverse primer: 5′-ATTACCGCGGCTGCTCCG-3′), *Akkermansia* (AM1; forward primer: 5′-CAGCACGTGAAGGTGGGGAC-3′, AM2; reverse primer: 5′-CCTTGCGGTTGGCTTCAGAT-3′), *Bacteroides* (AllBac296f; forward primer: 5′-GAGAGGAA GGTCCCCCAC-3′, AllBac412r; reverse primer: 5′-CGCTAC TTGGCTGGTTCAG-3′), *Butyricimonas* (Buty1f; forward primer: 5′-GGTGAGTAACACGTGTGCAAC-3′, Buty1r; reverse primer: 5′-TACCCCGCCAACTACCTAATG-3′), and *Mucispirillum* (MucisF; forward primer: 5′-CGTTTGCAAGAA TGAAACTCAAA-3′, MucisR; reverse primer: 5′-CACAGCATTATCTCTAACGCCTT-3′) were used for amplification (Layton et al., 2006; Collado et al., 2007; Cahenzli et al., 2013).

### Fecal Microbiota Transplantation (FMT)

Fecal material (0.1 g) from rosuvastatin-treated mice (HFD-Rosu group) was pooled in 1 mL of PBS. Mice were allowed to drink water containing penicillin G procaine (2,000 IU/L) and streptomycin (2.5 mg/L) for 5 days prior to FMT. After centrifugation at 2,000 *g* for 2 min, 500 μL of supernatant was administered to antibiotic-treated mice fed a HFD for 48 weeks (HFD-fRosu, *n* = 6) *via* oral gavage. Metabolic profiles, inflammatory cytokines and bacterial abundances were analyzed and compared between the RD (*n* = 5) and HFD (*n* = 4) groups 4 weeks after FMT.

### Statistical Analysis

Data for each group are presented as the mean ± standard error of mean (SEM). Relative abundance was analyzed using LEfSe based on the Kruskal–Wallis and Wilcoxon tests, and significance was defined at *P* < 0.05 (alpha value = 0.05). The logarithmic LDA score threshold was set at 3.0. To quantify *in vivo* mRNA levels relative to an internal control (GAPDH), the 2^–Δ^ ^Δ^ ^Ct^ relative quantification method (ΔΔCt = (C_t__.Target_ − C_t__.__β__–actin_)_*Group*__1_ − (C_*t*__.Target_ − C_t__.__β__–actin_)_Group__2_) was used. Statistical significance was assessed by one-way analysis of variance (ANOVA), followed by Duncan’s *post hoc* test with agricolae package in RStudio. All statistical analyses were performed using RStudio. A *P* value **<** 0.05 was considered to indicate statistical significance.

## Results

### Effects of Statins on Metabolic Improvements

**FIGURE 1:**
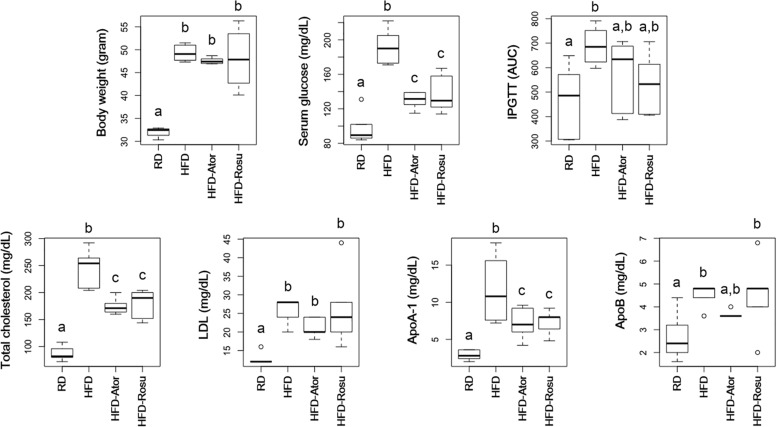
Effect of statins on body weight, serum glucose, intraperitoneal glucose tolerance test (IPGTT), total cholesterol, LDL, ApoA-1, and ApoB. Five-week-old C57BL/6N mice were fed a HFD for 39 weeks to induce metabolic disorders, and then statins were administered daily for 16 weeks. RD: regular diet (*n* = 6); HFD: high-fat-diet (*n* = 6); HFD-Ator: atorvastatin administration during HFD feeding (*n* = 6); HFD-Rosu: rosuvastatin administration during HFD feeding (*n* = 6). Different superscript letters indicate significant differences (*P* < 0.05) according to Duncan’s *post hoc* test following ANOVA.

As expected, both atorvastatin and rosuvastatin significantly reduced total cholesterol levels (Figure 1). Moreover, statins significantly reduced serum glucose levels and tended to improve glucose tolerance (Figure 1). In addition, statins decreased levels of ApoA-1 but not ApoB. There was no significant change in body weight and levels of LDL in response to statins compared to the corresponding values in the HFD group.

### Effects of Statins on the Gut Microbiota

**FIGURE 2:**
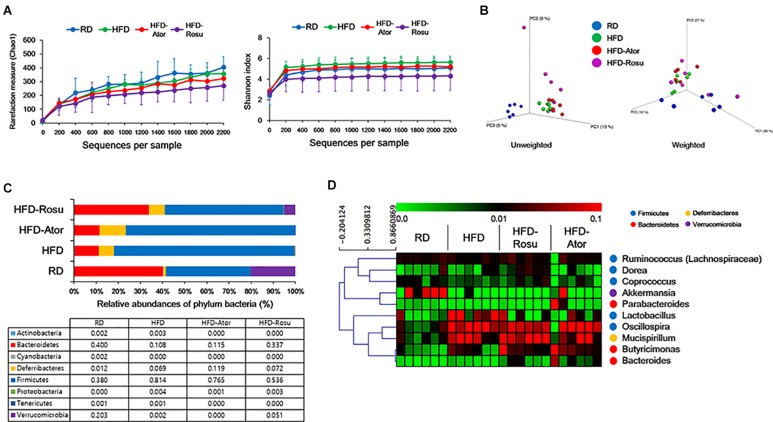
Microbial diversity according to diet and statin therapy. **(A)** Rarefaction curve and Shannon index of bacterial diversity. **(B)** PCoA of UniFrac distances. **(C)** Bacterial classification at the phylum level. Table value indicates relative abundance of phylum level. **(D)** Heatmap of average linkage hierarchical clustering of the top 10 most abundant bacterial genera using Spearman’s rank correlation. A heat map was generated using MultiExperiment Viewer (MEV) software (v4.8.1). Rows represent bacterial genera, and columns represent samples.

A total of 334,356 sequences were generated from 24 samples. An average of 13,932 ± 6,137 sequences were recovered per sample and used for comparative analyses. Figures 2A,B show the differences in microbial diversity between the RD, HFD, HFD-Ator, and HFD-Rosu groups. The alpha diversities of gut microbiota analyzed using Chao 1 richness and Shannon index did not reveal any significant difference between the groups (Figure 2A). The Principal coordinates analysis (PCoA) of UniFrac distances showed a separation between the RD, HFD, HFD-Ator, and HFD-Rosu groups, which was clearly classified in the unweighted PCoA (Figure 2B).

### Effects of Statins and Diet on the Composition of the Gut Microbiota

The phyla *Bacteroidetes* and *Firmicutes* from the RD group consisted of 40.0 ± 19.6 [average of relative abundance (%) ± standard deviation] and 38.0 ± 16.0% of gut microbiota, respectively, and the *Firmicutes/Bacteroidetes* (F/B) ratio was 1.3 ± 1.1%. The abundance of *Bacteroidetes* (10.8 ± 9.3%) decreased in the HFD group and that of *Firmicutes* (81.4 ± 13.4%) increased compared to the corresponding abundances in the RD group. The F/B ratio was 24.5 ± 28.6%. In contrast, the abundance of *Bacteroidetes* and *Firmicutes* from the HFD-Ator group was 11.5 ± 9.9% and 76.5 ± 10.6%, respectively; the F/B ratio was 10.6 ± 6.4%; and the abundance of *Bacteroidetes* and *Firmicutes* in the HFD-Rosu group was 33.7 ± 29.1% and 53.6 ± 24.9%, respectively. The F/B ratio was 2.8 ± 1.9%. These results indicate that rosuvastatin effectively restores the altered gut microbiota by HFD compared to atorvastatin. In addition, the abundance of *Deferribacteres* in the HFD-Ator group was 11.9 ± 4.4%, which was greater than that in the RD (1.2 ± 0.9%) and HFD (6.9 ± 4.4%) groups (Figure 2C). Relative abundances of *Bacteroidetes*, *Firmicutes*, and *Deferribacteres* in the HFD-Ator and HFD-Rosu group were not significantly different compared to the HFD group.

**FIGURE 3:**
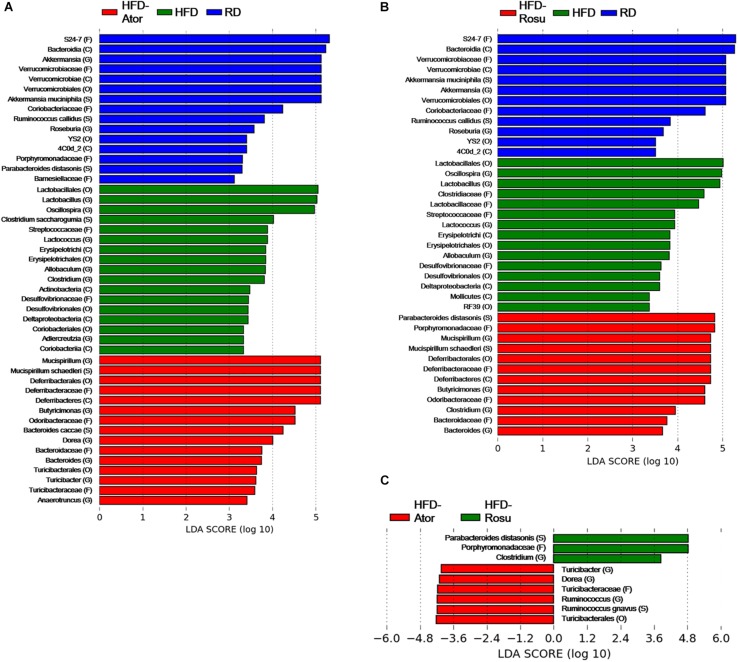
Significant differences in bacterial abundance according to diet and statin therapy. **(A)** Comparison between RD, HFD, and HFD-Ator groups. **(B)** Comparison between RD, HFD, and HFD-Rosu groups. **(C)** Comparison between HFD-Ator and HFD-Rosu groups. Significant differences were identified by LEfSe analysis at *P* < 0.05 by the Kruskal–Wallis test (among classes) and Wilcoxon test (between subclasses). The threshold logarithmic LDA score was 3.0. Three and four bacterial families were identified by atorvastatin and rosuvastatin, respectively. Among them, *Odoribacteraceae*, *Deferribacteraceae*, and *Bacteroidaceae* overlapped. At the genus level, *Bacteroides*, *Butyricimonas*, and *Mucispirillum* were increased by both atorvastatin and rosuvastatin.

Among the top 10 most abundant bacterial genera found in the four groups, *Bacteroides* and *Butyricimonas* of the phylum *Bacteroidetes*, *Oscillospira* of the phylum *Firmicutes*, and *Mucispirillum* of the phylum *Deferribacteres* were closely clustered (Figure 2D). Their abundance was enriched in the HFD-Ator and HFD-Rosu groups compared to that in the RD and HFD groups, accounting for 24.3 ± 5.6% and 27.8 ± 14.3% of the total identified bacterial OTUs, respectively (Figure 2D). In the LEfSe analysis, the abundances of *Anaerotruncus*, *Bacteroides*, *Butyricimonas*, *Dorea*, *Mucispirillum*, and *Turicibacter* were significantly greater in the HFD-Ator group than in the RD and HFD groups, and the abundances of *Bacteroides*, *Butyricimonas*, *Clostridium*, and *Mucispirillum* were significantly greater in the HFD-Rosu group. The abundances of *Bacteroides*, *Butyricimonas*, and *Mucispirillum* overlapped between the HFD-Ator and the HFD-Rosu groups (Figures 3A,B). Moreover, in comparison between the HFD-Ator and the HFD-Rosu groups, the abundances of *Turicibacter*, *Dorea*, and *Ruminococcus* were significantly greater in the HFD-Ator group than in the HFD-Rosu group, and the abundance of *Clostridium* was significantly greater in the HFD-Rosu group than in the HFD-Ator group (Figure 3C).

Among them, *Odoribacteraceae*, *Deferribacteraceae*, and *Bacteroidaceae* overlapped. At the genus level, *Bacteroides*, *Butyricimonas*, and *Mucispirillum* were increased by both atorvastatin and rosuvastatin.

### Effects of Statins on Inflammatory Cytokines

**FIGURE 4:**
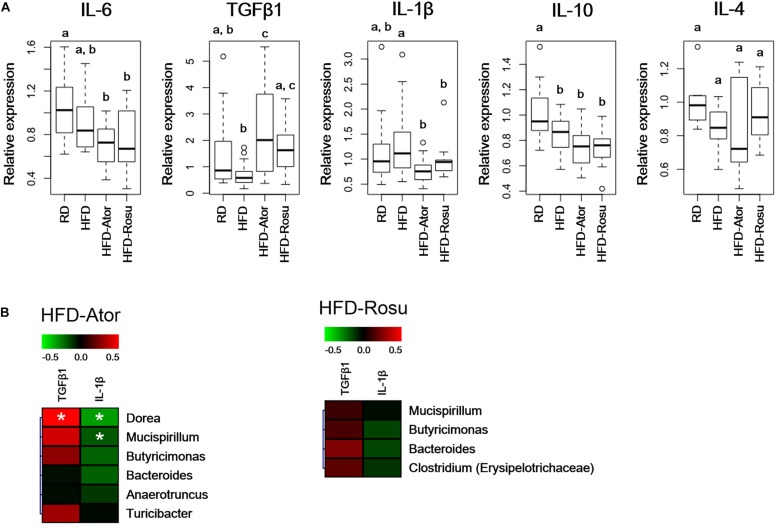
Relative expression levels of inflammatory cytokines in the ileum. Relative mRNA levels of cytokines were analyzed by qPCR. **(A)** Relative expression levels of IL-6, TGFβ1, IL-1β, IL-10, and IL-4. Different superscript letters indicate significant differences (*P* < 0.05) according to Duncan’s *post hoc* test following ANOVA. **(B)** Correlations between immunological biomarkers and the abundance of bacterial genera in the HFD-Ator (*n* = 6) and HFD-Rosu group (*n* = 6). Statistical significance (Spearman’s correlation coefficient; *P* < 0.05).

TGFβ1 expression increased significantly in the HFD-Ator and HFD-Rosu groups compared to that in the HFD group, whereas IL-1β expression decreased with statin treatments. There was no significant change in the expression of IL-4, IL-6, and IL-10 by statins compared to that in the HFD group (Figure 4A). In addition, the expression of TGFβ1 and IL-1β was correlated with increased abundance of the *Clostridium*, *Dorea*, *Mucispirillum*, *Butyricimonas*, *Bacteroides*, *Anaerotruncus*, and *Turicibacter* by statins. Among them, the positive correlation between TGFβ1 and the abundance of *Dorea* and the negative correlation between IL-1β and the abundance of *Dorea* and *Mucispirillum* were significant in the HFD-Ator group (Figure 4B).

### Effects of Fecal Material Transplantation on Metabolic Improvements

**FIGURE 5:**
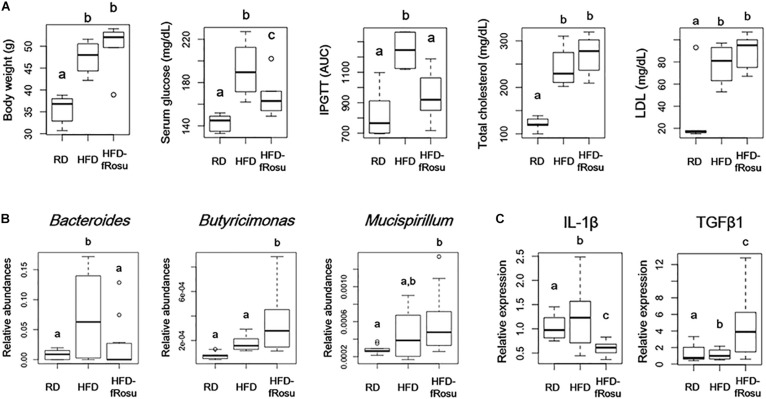
Effect of fecal microbiota transplantation (FMT) on metabolic parameters, gut microbiota, and inflammatory cytokines. Six-week-old mice were fed a HFD for 43 weeks and then inoculated orally with pooled fecal material from the HFD-Rosu group for 4 weeks (HFD-fRosu, *n* = 6). All parameters were compared to those in the RD (*n* = 5) and HFD (*n* = 4) groups. **(A)** The effect of FMT on body weight, serum glucose, IPGTT, total cholesterol, and LDL. **(B)** Abundance of *Bacteroides*, *Butyricimonas*, and *Mucispirillum*. Relative abundance was analyzed using qPCR. **(C)** Relative expression levels of IL-1β and TGFβ1 in the ileum. Relative mRNA levels were analyzed using qPCR. Different superscript letters indicate significant differences (*P* < 0.05) according to Duncan’s *post hoc* test following ANOVA.

Both atorvastatin and rosuvastatin significantly improved the metabolic disorders, and microbial diversity of the rosuvastatin-treated mice group was more different compared to the HFD group than the atorvastatin-treated mice group based on beta diversity. Therefore, fecal material transplantation (FMT) was performed using fecal material from rosuvastatin-treated mice, which significantly improved serum glucose levels and glucose tolerance with the HFD (Figure 5A). However, body weight and total cholesterol and LDL levels were not improved by FMT. The abundances of *Bacteroides*, *Butyricimonas*, and *Mucispirillum* were 2.2 ± 4.0%, 0.04 ± 0.02%, and 0.06 ± 0.03% in the HFD-fRosu group, respectively. Among them, the abundance of *Bacteroides* was significantly decreased and that of *Butyricimonas* was significantly increased after FMT compared to that in the HFD group (Figure 5B). TGFβ1 expression increased significantly, whereas IL-1β expression decreased significantly, in the HFD-fRosu group compared to that in the HFD group (Figure 5C).

## Discussion

Modulation of gut microbiota has an impact on metabolic improvements in obesity and T2D (Clarke et al., 2012; Mardinoglu et al., 2016). Recent studies have revealed that certain pharmacotherapies can improve metabolic diseases through specific compositional changes in gut microbiota (Shin et al., 2013; Lee and Ko, 2014). Statins are cholesterol-lowering drugs, which also affect glucose tolerance in animal models. The characteristics of gut microbiota in response to statins have not yet been fully investigated in metabolic diseases (Caparros-Martin et al., 2017; Khan et al., 2018). In this study, changes in gut microbiota in response to atorvastatin and rosuvastatin were observed in mice with HFD-induced obesity, and the abundances of *Bacteroides, Butyricimonas*, and *Mucispirillum* were all remarkable. Moreover, FMT with fecal materials from the HFD-Rosu group has an impact on hyperglycemia improvement, which may be related to the abundance of *Butyricimonas*.

Therapy with atorvastatin and rosuvastatin both induced significant changes in the gut microbiota during the HFD. In a meta-analysis comparing the two statins, similar pharmacokinetics, efficacy, and side effects were observed, which may be associated with the similar changes in gut microbiota induced by atorvastatin and rosuvastatin (Abbas et al., 2012). Nevertheless, differences in gut bacterial composition were observed with atorvastatin and rosuvastatin, which may be caused by differences in chemical structure and the effects on hyperlipidemia and glucose metabolism. Atorvastatin is lipophilic and rosuvastatin is hydrophilic due to the side chain of the methane sulfonamide group, and across the dose range used, rosuvastatin was significantly more effective in decreasing cholesterol than atorvastatin. However, it is unclear how those differences influence gut microbiota.

Alterations in gut microbiota in recent studies were not consistent with the findings of the present study (Nolan et al., 2017; Khan et al., 2018), which may be due to two major differences in the experimental method used. The first is due to differences in the target region of the 16S rRNA gene. In NGS analysis, the target region of the 16S rRNA gene impacts the microbiota results (Rintala et al., 2017). Results of this study are based on PCR amplification and sequencing targeting V4 region, and a different short variable region was targeted in different previous studies. Consequently, that affected the universality of the NGS result, which is why amplification of multiple variable regions is recommended to increase the universality and resolution (Fuks et al., 2018). Second, although both studies were performed using C57BL/6 mice, the gender and age of the mice were different. The effect of medication on the gut microbiota is influenced by gender (Lee and Ko, 2014). Liu et al. (2018) reported that variation in gut microbiota was associated with the efficacy of rosuvastatin therapy in a clinical study, with differences in gut microbiota observed by age and gender (Liu et al., 2018). In the present study, statins were administered for 16 weeks in 29-week-old aged male mice; therefore, our microbiota results may reflect the characteristics of gut microbiota in response to statin therapy. A decrease in the F/B ratio was observed with the metabolic improvement induced by statins treatment. *Bacteroidetes* and *Firmicutes* are the major bacterial phyla, which represent most bacteria in the gut, and their ratio is related with obesity-related metabolic disorders (Ley et al., 2006; Chakraborti, 2015; Louis et al., 2016). Moreover, in a recent study, statin therapy with pravastatin and atorvastatin resulted in gut dysbiosis in HFD-fed female C57BL/6 mice, which was associated with adverse effects induced by statins intolerance, including myopathy and T2D (Caparros-Martin et al., 2017). The results of recent studies suggest that statins influence changes in gut microbiota and that various factors affect the composition of gut microbiota, including age, gender, and diet. Nevertheless, recent studies reported that statin therapy increased butyric acid-producing bacteria, including those belonging to the families *Lachnospiraceae*, *Bacteroidaceae*, and *Prevotellaceae*. The genera *Bacteroides* and *Butyricimonas*, which were increased in this study, are butyric acid-producing bacteria, and short-chain fatty acids (SCFAs) may play key roles in metabolic improvements, including hyperlipidemia and hyperglycemia.

SCFAs are produced from dietary fiber by intestinal bacteria, and this has beneficial effects on host energy metabolism (Den Besten et al., 2013). Among the SCFAs, acetic acid, propionic acid, and butyric acid are most abundant in the intestine, representing over 90% of the SCFAs present (Den Besten et al., 2013). A high abundance of *Bacteroides* spp. is characteristic of non-obese individuals and is caused by the releases of propionic acid and acetic acid from *Bacteroides* spp. (Chakraborti, 2015). Another SCFA, butyric acid, improves the outcome of insulin resistance and dyslipidemia (Gao et al., 2009). Several bacterial strains including *Clostridium butyricum* and *Butyricicoccus pullicaecorum* are representative butyric acid-producing anaerobic bacteria, which are involved in T2D and inflammatory bowel disease, respectively (Geirnaert et al., 2014; Jia et al., 2017). Additionally, the majority of members of the *Prevotellaceae*, *Clostridiaceae*, *Ruminococcaceae*, *Lactobacillaceae*, and *Lachnospiraceae* families are putative butyric acid producers and have anti-inflammatory properties (Esquivel-Elizondo et al., 2017). In the present study, *Butyricimonas*, a butyric acid-producing bacterium, may have played a key role in metabolic improvements induced by statins, especially in glucose metabolism. Moreover, FMT using fecal materials from the HFD-Rosu group resulted in improved glucose tolerance, and the high abundance of *Butyricimonas* was solely maintained. However, butyric acid was not investigated in this study, and the mechanisms through which *Butyricimonas* induce metabolic improvements are unknown. This limitation should be addressed in future studies.

Apart from butyric acid-producing bacteria, *Mucispirillum*, which inhabits the intestinal mucus layer, may have an important role in the metabolic improvements induced by statins. To date, few studies have discussed how *Mucispirillum schaedleri* are associated with intestinal inflammation (Loy et al., 2017). However, the role of *Mucispirillum* in lipid metabolism and inflammation is poorly understood. In the present study, *Mucispirillum* was more abundant in the HFD group than in the RD group, and was significantly increased by statins with a HFD. Moreover, the abundance of those bacteria was negatively correlated with pro-inflammatory cytokine and positively correlated with anti-inflammatory cytokines in the statin-treated HFD group. Conversely, based on genome analysis, *M. schaedleri* is not predicted to be a primary degrader of glycan from mucin. Instead, *M. schaedleri* is likely to utilize SCFAs for energy metabolism (Loy et al., 2017); those abundant in this study may be accompanied by SCFA-producing bacteria in response to statins.

Regulation of IL-1β and TGFβ expression may play a key role in the metabolic improvements associated with statin therapy, due to changes in gut microbiota. A previous study reported that statin therapy regulates inflammatory cytokines such as IL-1β, IL-6, and TNF-α (Lee et al., 2016). In the present study, IL-1β and TGFβ expression increased in the ileum, indicating that the regulation of IL-1β and TGFβ is associated with the effect of statins on the alleviation of hyperglycemia, hyperlipidemia, and inflammation. In the intestinal immune system, the pro-inflammatory cytokine IL-1β plays an important role in insulin resistance and hyperlipidemia (Rotter et al., 2003; Fentoglu et al., 2011). Especially, upregulation of IL-1β is a general feature underlying obesity-related insulin resistance, which increases intestinal epithelial tight junction permeability and inflammation (Al-Sadi and Ma, 2007). Intestinal permeability has been observed in dysbiosis by HFD, and changes in gut microbiota in response to statin therapy with a HFD played an important role in the downregulation of IL-1β expression and the improvement of downstream glucose control (Winer et al., 2016). Secretion of TGFβ, an anti-inflammatory mediator, occurs under normal physiological conditions in the intestine; statin therapy increased TGFβ expression (Porreca et al., 2002). TGFβ is a pleiotropic cytokine involved in various immune signaling pathways, including intestinal anti-inflammation (Letterio and Roberts, 1998). Above all, TGFβ signaling suppresses T-cell proliferation and activation through Treg differentiation, which is essential for intestinal homeostasis (Bauche and Marie, 2017). In intestinal immunity, TGFβ suppresses inflammatory responses through the canonical pathway in a mechanism mediated by the intracellular signaling proteins SMAD2/3 (Massague, 2012). Interestingly, TGFβ production is induced by gut microbiota, and *Clostridium* strains including *Clostridium butyricum* (or derived butyric acid) and *Bacteroides fragilis* were reported to induce TGFβ production and Treg differentiation (Round and Mazmanian, 2010; Kashiwagi et al., 2015; Hong et al., 2017; Goncalves et al., 2018). Therefore, the anti-inflammatory effects of statin therapy may be associated with the regulation of TGFβ expression in the intestine *via* the modulation of gut microbiota.

In this study, the effect of gut microbiota on metabolic improvements, especially anti-hyperglycemic, was shown to be induced by FMT. After FMT, the relative abundance of *Butyricimonas* increased significantly, compared to that in the HFD group, which may play a key role in the anti-hyperglycemic effect. In a previous study, the increased abundance of *Butyricimonas* and *Bacteroides* with metformin therapy was associated with metabolic improvements, including body weight, glucose tolerance, and lipid metabolism, and the administration of fecal material from metformin-treated mice had an effect on body weight change only, which was associated with the abundance of *Bacteroides* (Lee H. et al., 2018). Additionally, a recent study reported that *Bacteroides* prevented obesity and insulin resistance (Yang et al., 2017). Along with previous studies, the present study indicates that *Butyricimonas* may have a more important role in hyperglycemia than in obesity. Moreover, the regulation of IL-1β and TGFβ1 by FMT was consistent with the effects of statin therapy during HFD. FMT is a therapeutic option for inflammatory bowel disease induced by *Clostridium difficile* infection, and recent studies have reported metabolic improvements, including insulin sensitivity, with FMT (Kootte et al., 2017). However, the mechanism underlying the effect of FMT on metabolism is unclear, and neither criteria for donor selection nor optimal processes were established. In this study, the anti-hyperglycemic effect persisted for 1 month after FMT in antibiotic-treated mouse model, which provides information for successful FMT therapy in metabolic diseases treatment.

## Conclusion

Statin therapy with atorvastatin and rosuvastatin significantly altered the gut microbiota during HFD in an aged obese mice model. The abundance of the genera *Bacteroides*, *Butyricimonas*, and *Mucispirillum* was significantly increased by statins, and this was related to hyperglycemia and hyperlipidemia. In particular, *Butyricimonas* may be related to the anti-hyperglycemic effect of statins. Furthermore, the downregulation of IL-1β and the upregulation of TGFβ1 by statins were significantly correlated with the abundance of those bacteria. These results suggest that the alterations in gut microbiota by statins may be one of therapeutic targets for the treatment of hyperglycemia.

## Data Availability

The datasets generated for this study can be found in FigShare https://figshare.com/articles/NGS_data/9104477.

## Ethics Statement

This study was approved by the Institutional Animal Care and Use Committee (IACUC) of Sahmyook University for the care and use of laboratory animals (SYUIACUC 2015001).

## Author Contributions

HL, HK, YS, and KK contributed to the study conception and design. HL, JK, and JA performed by the experiments. HL and JA performed the data analysis and interpretation of the data. HL and JA contributed to the manuscript drafting. All authors have approved the final version of this manuscript.

## Conflict of Interest Statement

The authors declare that the research was conducted in the absence of any commercial or financial relationships that could be construed as a potential conflict of interest.

## Footnotes

1 https://figshare.com/articles/NGS_data/9104477
